# Efficacy of obstructive sleep apnea treatment by antileukotriene receptor and surgery therapy in children with adenotonsillar hypertrophy: A descriptive and cohort study

**DOI:** 10.3389/fneur.2022.1008310

**Published:** 2022-09-27

**Authors:** Dien Tran-Minh, Anh Phi-Thi-Quynh, Phuc Nguyen-Dinh, Sy Duong-Quy

**Affiliations:** ^1^Department of ENT, National Pediatric Hospital, Hanoi, Vietnam; ^2^Department of ENT, Hanoi University of Medicine, Hanoi, Vietnam; ^3^Sleep Lab Center, Lam Dong Medical College and Bio-Medical Research Center, Dalat, Vietnam; ^4^Immuno-Allergology Division, Hershey Medical Center, Penn State Medical College, State College, PA, United States; ^5^Department of Outpatient Expert Consultation, Pham Ngoc Thach University of Medicine, Ho Chi Minh City, Vietnam

**Keywords:** adenotonsillar hypertrophy, OSA, apnea-hypopnea index, snoring, antileukotriene receptor, adenotonsillectomy

## Abstract

**Background:**

Prevalence of obstructive sleep apnea (OSA) in children with adenotonsillar hypertrophy is high and related to the occlusion of the upper airway. The main treatments of OSA in these children is adenotonsillectomy. However, this intervention is an invasive method with a various success rate. Thus, the indications of tonsillectomy remain debatable and non-invasive treatment is still a potential choice in these patients.

**Methods:**

It was a cross-sectional and interventional study. This study included children aged from 2 to 12 years-old who were diagnosed with OSA by respiratory polygraphy and had tonsillar hypertrophy with/without adenoid hypertrophy. All main data including age, gender, height, weight, body mass index (BMI), clinical symptoms, and medical history were recorded for analysis. Physical examination and endoscopy were done to evaluate the size of tonsillar and adenoid hypertrophy by using Brodsky and Likert classifications, respectively. The severity of OSA was done by using the classification of AHI severity for children.

**Results:**

There were 114 patients (2–12 years old) with a mean age of 5.5 ± 2.1 years included in the present study. The main reasons for consultations were snoring (96.7%), a pause of breathing (57.1%), an effort to breathe (36.8%), unrefreshing sleep (32%), doziness (28.2%), and hyperactivity (26.3%). There were 36% of subjects with tonsillar hypertrophy grade 1–2, 48.2% with grade 3, and 15.8% with grade 4 (Brodsky classification); among them, there were 46.5% of subjects with grades 1–2 of adenoid hypertrophy, 45.6% with grade 3, and 7.0% with grade 4 (Likert classification). The mean AHI was 12.6 ± 11.2 event/h. There was a significant correlation between the mean AHI and the level of tonsillar and adenoid hypertrophy severity (r = 0.7601 and r = 0.7903; *p* < 0.05 and *p* < 0.05, respectively). The improvement of clinical symptoms of study subjects was found in both groups treated with ALR (antileukotriene receptor) or ST (surgery therapy). The symptoms related to OSA at night including snoring, struggle to breathe, sleeping with the mouth open, and stopping breathing during sleep were significantly improved after treatment with ATR and with ST (*p* < 0.001 and *p* = 0.001, respectively). The mean AHI was significantly reduced in comparison with before treatment in study subjects treated with ALR (0.9 ± 1.0 vs. 3.9 ± 2.7 events/h; *p* = 0.001) or with ST (3.5 ± 1.4 vs. 23.4 ± 13.1 events/h; *p* < 0.001).

**Conclusion:**

The treatment of OSA due to adeno-tonsillar hypertrophy with ALR for moderate OSA or surgery for severe OSA might reduce the symptoms related to OSA at night and during the day.

## Introduction

Obstructive sleep apnea (OSA) is a complete or partial obstruction of the upper airway during sleep and leads to intermittent hypoxia, the creation of oxidative stress, and fragmented sleep (1, 2). OSA can be found in adults, children, and infants (3). The prevalence of OSA in childhood is ~1–4% and depends on diagnostic criteria (4). Significantly, the prevalence of pediatric OSA has two peak periods. The first peak occurs in children between 2 and 8 years with enlarged tonsils and adenoids. The second peak appears during adolescence with weight gain (5).

OSA can lead to dire health consequences and a significant economic burden without prompt diagnosis and treatment (6). Children with OSA can have central nervous system disturbances such as attention deficit hyperactivity, depression, lack of concentration, and excessive daytime sleepiness (7). In addition, children with OSA have reported for risk of long-term cardiovascular consequences, including hypertension, arrhythmia, abnormal ventricular morphology, impaired ventricular contractility, and elevated right heart pressure (8, 9). Evenly, OSA can lead to death in children, especially sudden death at night (10).

The choice of OSA treatment will depend upon age, clinical symptoms, comorbidities, risk factors, and polysomnography (PSG) results (11). Because in children, tonsillar and adenoid hypertrophy is a major cause of OSA, most children can be treated with surgical adenotonsillectomy (12). However, children reveal a high risk for postoperative complications and the effects of long-term tonsillectomy on the immune system (13, 14). Therefore, pharmacologic therapy can be considered to initiate mild to moderate OSA. Interestingly, tonsillar tissue from children with OSA was reported to overexpress CysLT1, so some RCT applied montelukast for treatment. These reports showed improvement in the severity of OSA and adenoidal hypertrophy in children with non-severe OSA (15–17).

Therefore, the present study was conducted to evaluate the clinical efficacy of antileukotriene drugs and adenotonsillectomy in OSA children with tonsillar hypertrophy.

## Methods

### Subjects

There were 114 children from 3 to 12 years old included in the present study from August 2016 to December 2019 in National Pediatric Hospital in Vietnam. The present study was approved by the Ethical Committee of Hanoi Medical University and National Pediatric Hospital (No 99/HDDD-DHYHN).

#### Inclusion criteria

Children having all the following criteria were included in the present study: tonsillar hypertrophy, OSA defined by AASM (American Academic of Sleep Medicine), aged from 3 to 12 years old, and agreement from patients and their guardians.

#### Exclusion criteria

Children having one of the following criteria were excluded from the study: cranial-facial abnormal structure, Down syndrome, Pierre-Robin syndrome, Treacher Collin syndrome, micro crania, other disorders of the upper airway, neuro-muscular junction disorder such as myasthenia gravis, coagulopathy, renal failure, heart failure, or disagreement from patients and their guardians. All children currently treated with corticosteroids (oral, inhaled or intranasal form) or antihistamines (oral form) or nasal decongestants were also excluded from the study. Patients under antileukotriene therapy with increasing symptoms and refusing surgical treatment were also excluded from the present study.

### Methods

#### Study design

**Figure 1:**
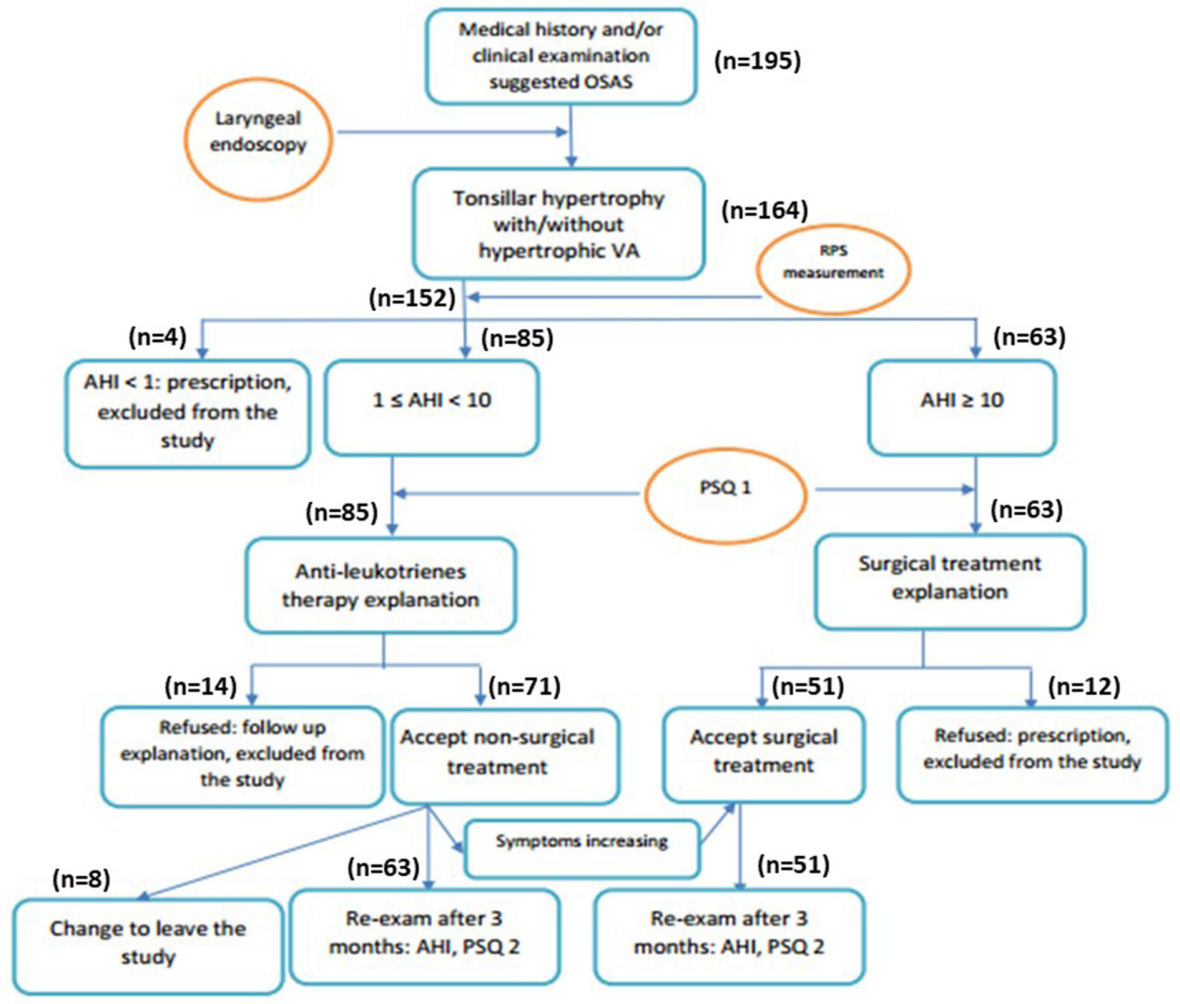
Flow-chart of study process. AHI, apnea-hypopnea index; PSQ, pediatric sleep quality; RPS, respiratory polysomnography.

It was a cross-section study; all study subjects with tonsillar hypertrophy received medical treatment if they had a mild-moderate OSA (Group 1) or surgical treatment if they had severe OSA (Group 2); those who were unresponsive to medical treatment after 1 month also received surgical treatment (Group 3; Figure 1). To avoid the bias, OSA children with comorbidities or who were not adherent to antileukotriene therapy during follow-up (monthly) were also excluded from the study. The compliance of antileukotriene therapy was evaluated monthly for each study subject.

#### Respiratory polygraphy

OSA was defined with polygraphy by using the apnea – hypopnea index (AHI) to classify the severity of OSA as recommended: normal (non-OSA): AHI ≤ 1/h; mild OSA: 1/h <AHI ≤ 5/h; moderate OSA: 5/h <AHI ≤ 10/h; severe OSA: AHI > 10/h (18, 19). Polygraphy was done with Apnea Link (ResMed; San Diego, California, USA).

#### Tonsillar and adenoid hypertrophy evaluation

Tonsillar hypertrophy was defined by using Brodsky's grading scale (20). There are 5 levels of tonsil hypertrophy based on the ratio of tonsil to the pharynx (distance between two anterior pillars), including grade 0 (located in the cavity), grade 1: occupied <25% of the distance between the two anterior pillars, grade 2 /3/ and 4: occupied 25–50%/ 50–75%/ and > 75% of the distance between the two anterior pillars, respectively.

Adenoid hypertrophy was defined by Likert's classification (21). There are 5 levels of adenoid hypertrophy based on the occlusion of posterior nasal aperture, including grade 1: occluded from 0 to 25%, grade 2: occluded from 25 to 50%, grade 3: occluded from 50 to 75%, and grade 4: occluded > 75% of posterior nasal aperture.

#### Data collection

All data on age, gender, height, weight, BMI, medical and family history, clinical characteristics, PSQ (Pediatric Sleep Questionnaire) scores, Mallampati classification, SSS (snoring severity scale) scores, and PG parameters (AHI, SpO2, pulse, and frequency of snoring) of the study subjects were collected for statistical analyses.

#### Statistical analysis

Epidata and Stada 15 were used to analyze the recorded data. Continuous variables were presented as mean ± standard deviation (SD). Skewness-Kurtosis test was used for evaluating the normal distribution and Kruskal–Wallis test was done for performing the pairwise comparison. Multiple regression analysis was performed to measure the correlation between AHI and continuous variables with coefficient R of Pearson for normal distribution or Spearman for non-normal distribution variables.

## Results

### Clinical characteristics and respiratory features of study subjects

There were 114 patients (2–12 years old) with a mean age of 5.5 ± 2.1 years included in the present study. The gender rate was 3.1/1 (male/female) (Table 1A). The percentage of subjects in the age group of 3–8 years old was 75.5% and underweight was 27.2%. For medical history, there was 33.2% of patients had allergic rhinitis, 11.2% of asthma, and 68.4% with a family history of snoring (Table 1A).

**Table 1A:** Clinical characteristics and respiratory polygraphy features of study subjects.

**Parameters**	**Study subjects (*****n*** = **114)**		**Parameters**	**Study subjects (*****n*** = **114)**	
	**Mean ±SD**	***n* (%)**		**Mean ±SD**	***n* (%)**
Age, years	5.5 ± 2.1	-	**Tonsillar hypertrophy**
BMI, kg/m^2^	16.9 ± 3.2	-	Grade 1–2	-	41 (36.0)
**Gender**			Grade 3	*-*	55 (48.2)
Male	-	75.4	Grade 4	-	18 (15.8)
Female	-	24.6	**Adenoid hypertrophy**
**Main reason of consultation**			Grade 1–2	*-*	54 (46.5)
Snoring	-	96.7	Grade 3	-	52 (45.6)
Pause of breathing	-	57.1	Grade 4	-	8 (7.0)
Effort to breath	-	36.8	**Respiratory polygraphy**
Unrefreshing sleep	-	32.0	AHI (event/h)	12.6 ± 11.2	-
Doziness	-	28.2	Mild	-	48 (42.1)
Hyperactivity	-	26.3	Moderate	-	22 (19.3)
Loss of concentration	-	17.5	Severe		44 (38.6)
Daytime sleepiness	-	14.9	SpO2 in the OSA period	80.6 ± 12.8	-
Nasal congestion at night	-	13.2	Nadir SpO2	75.7 ± 13.7	-
Wake up during sleep	-	12.3	Number event of snoring	426.3 ± 315.9	-

BMI, body mass index; AHI, apnea-hypopnea index.

The main reasons for consultations were snoring (96.7%), a pause of breathing (57.1%), an effort to breathe (36.8%), unrefreshing sleep (32%), doziness (28.2%), hyperactivity (26.3%), loss of concentration (17.5%), daytime sleepiness (14.9%), nasal congestion nose at night (13.2%), and wake up during sleep (12.3%) (Table 1A).

ENT examination showed that 36% of subjects with tonsillar hypertrophy grade 2, 48.2% with grade 3, and the most common age was from 3 to 8 years old (75.4%). There were 46.5% of subjects with grades 1–2 of adenoid hypertrophy and 45.6% with grade 3 (Table 1A). The classification of tonsillar and adenoid hypertrophy severity was presented in Table 1B.

**Table 1B:** The classification of tonsillar hypertrophy and adenoid hypertrophy by age group.

**Age group**	**Tonsillar hypertrophy severity**			**Total** ***N* (%)**	**Adenoid hypertrophy severity**			**Total** ***N* (%)**
	**Grade 1+2** **N (%)**	**Grade 3** **N (%)**	**Grade 4** **N (%)**		**Grade 1+2** **N (%)**	**Grade 3** **N (%)**	**Grade 4 N (%)**	
<3 years	3 (18.8)	10 (62.5)	3 (18.8)	16 (100.0)	4 (25.0)	8 (50.0)	4 (25.0)	16 (100.0)
3–5 years	6 (18.8)	20 (62.5)	6 (18.8)	32 (100.0)	12 (37.5)	18 (56.2)	2 (6.3)	32 (100.0)
5–8 years	23 (42.6)	23 (42.6)	8 (14.8)	54 (100.0)	27 (50.0)	25 (46.3)	2 (3.7)	54 (100.0)
>8 years	9 (75.0)	2 (16.7)	1 (8.3)	12 (100.0)	11 (91.7)	1 (8.3)	0 (0.0)	12 (100.0)
Total	41 (36.0)	55 (48.2)	18 (15.8)	114 (100.0)	54 (46.5)	52 (45.6)	8 (7.0)	114 (100.0)

**Figure 2:**
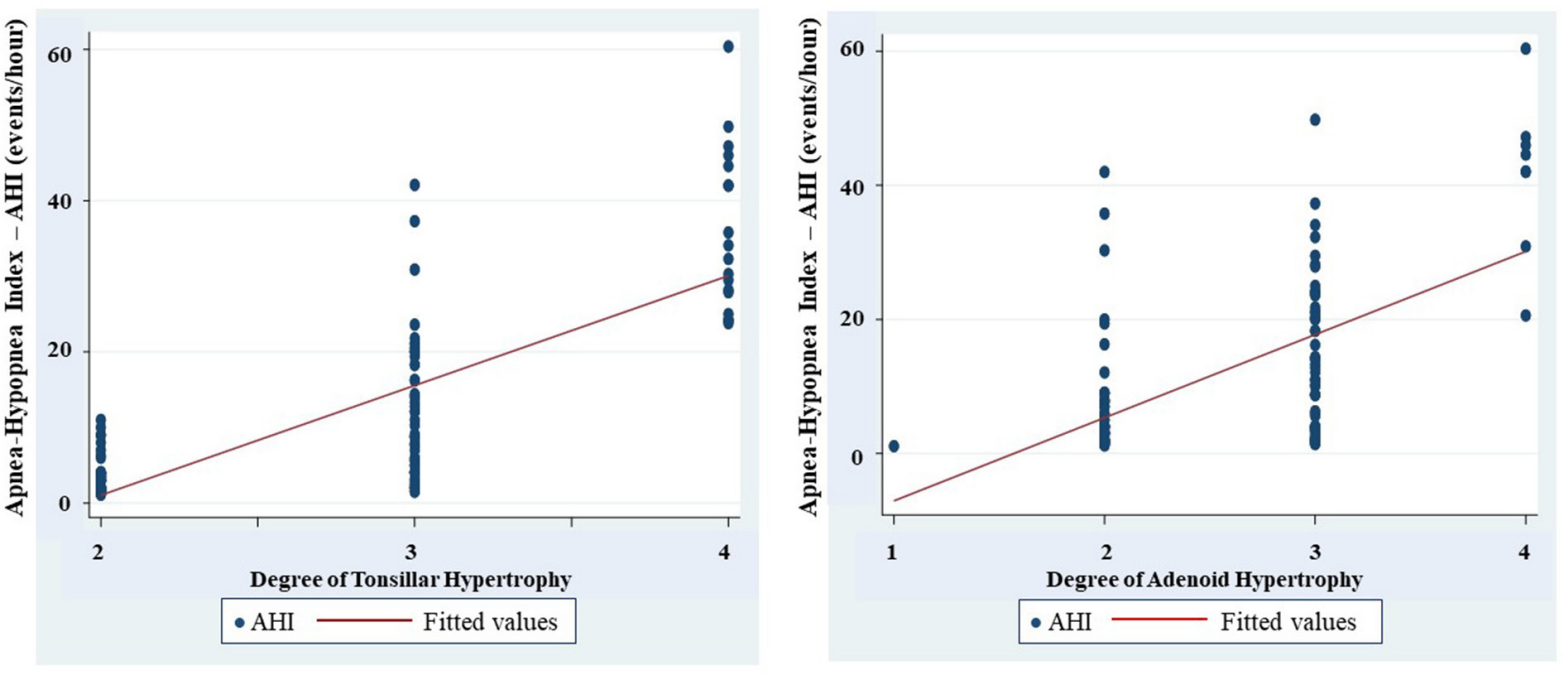
Correlation between degree of tonsilar and adenoid hypertrophy and apnea-hypopnea index (AHI).

Respiratory polygraphy of study subjects showed that the average apnea hypopnea index (AHI) was 12.6 ± 11.2 event/h; the lowest oxygen saturation was 75.7 ± 13.7%, and the number of events of snoring was 426.3 ± 315.9 (Table 1A). There was an increasing and significant correlation between the mean AHI and the level of tonsillar and adenoid hypertrophy severity (r = 0.7601 and r = 0.7903; *p* < 0.05 and *p* < 0.05, respectively; Figure 2).

### Characteristics of study subjects classified by treatments

The results showed that subjects treated with surgical therapy (ST) were younger and had higher BMI than those treated with anti-leukotriene receptors (ALR) (4.9 ± 1.9 and 17.7 ± 3.6 vs. 5.9 ± 2.1 years and 16.4 ± 2.8 kg/m^2^; *p* = 0.004 and *p* = 0.030; respectively; Table 2). There was no significant difference in gender between the two groups (Table 2).

**Table 2:** Anthropometric and respiratory polygraphy characteristics of study subjects classified by treatments.

**Parameters**	**Total study** **subjects** **(*n* = 114)**	**Subjects treated with ST** **(*n* = 51)**	**Subjects treated with ALR** **(*n* = 63)**	p*
**Anthropometry**
Age	5.5 ± 2.1	4.9 ± 1.9	5.9 ± 2.1	0.004
BMI	16.9 ± 3.2	17.7 ± 3.6	16.4 ± 2.8	0.030
**Gender**	-	-	-	-
Male, %	75.4	82.4	69.8	0.063
Female, %	24.6	17.6	30.2	0.123
**Respiratory polygraphy**
AHI (event/h), mean ± SD	12.6 ± 11.2	23.4 ± 13.1	3.9 ± 2.7	<0.001
Mild, *N* (%)	48 (42.1)	1 (2.0)	47 (74.6)	<0.001
Moderate, *N* (%)	22 (19.3)	6 (11.8)	16 (25.4)	<0.001
Severe, *N* (%)	44 (38.6)	44 (86.2)	0 (0.0)	<0.001
SpO2, mean ± SD (%)	95.2 ± 5.6	94.0 ± 7.7	96.3 ± 2.4	0.008
SpO2 in the OSA period, mean ± SD (%)	80.6 ± 12.8	74.2 ± 13.9	86.0 ± 8.8	0.001
Nadir SpO2, mean ± SD (%)	75.7 ± 13.7	71.9 ± 14.9	78.8 ± 11.9	0.012
Average pulse, mean ± SD (%) (p/m)	87.7 ± 16.5	93.8 ± 18.7	82.7 ± 12.6	0.001
Snoring, mean ± SD (%) (event)	426.3 ± 315.9	633.6 ± 433.2	276.6 ± 257.3	0.054

BMI, body mass index; AHI, apnea-hypopnea index; SG, surgical therapy; ALR, anti-leukotriene receptor. ^*^Subjects with ALR vs. SG.

The mean AHI of study subjects treated with ST for adenotonsillar hypertrophy was higher than that in study subjects treated with ALR (23.4 ± 13.1 vs. 3.9 ± 2.7 and <0.001; Table 2). The percentage of severe OSA in study subjects treated ST group was significantly higher than those treated with ALR (86.2 vs. 0.0%; *p* < 0.001). There was 74.6% of mild OSA and 25.4% of moderate OSA in study subjects treated with ALR (Table 2). The mean SpO2 in the OSA period of subjects treated with ST was significantly lower than those treated with ALR (74.2 ± 13.9% vs. 86.0 ± 8.8% and *p* = 0.001. There were not any significant differences in nadir SpO2 and snoring events between the two groups (Table 2).

### Clinical improvements of treatments in study subjects

The improvement of clinical symptoms of study subjects with OSA was found in both groups of treatment with ALR and ST (Table 3). The symptoms related to OSA at night including snoring, struggle to breathe, sleeping with the mouth open, and stopping breathing during sleep were significantly improved after treatment with ATR and with ST (*p* < 0.001 and *p* = 0.001, respectively; Table 3).

**Table 3:** Modification of clinical symptoms and respiratory polygraphy after treatment.

**Symptoms**	**Study subjects treated with ALR (*****n*** = **63)**						**Study subjects treated with ST (*****n*** = **51)**					
	**Pre**		**Post**		**Δ**	p*	**Pre**		**Post**		**Δ**	p*
	** $\bar{X}$ **	**SD**	** $\bar{X}$ **	**SD**			** $\bar{X}$ **	**SD**	** $\bar{X}$ **	**SD**		
**Symptoms at night**												
Snoring	3.1	0.4	1.2	0.4	1.87	<0.001	3.6	0.5	1.0	0.5	2.59	0.001
Snoring loudly	2.2	0.9	0.7	0.6	1.59		3.3	0.6	0.6	0.5	2.72	
Struggle to breath	1.9	0.8	0.3	0.2	1.68		2.8	0.9	0.4	0.3	2.39	
Sleep with opening mouth	2.2	0.7	0.7	0.5	1.48		3.3	0.5	0.5	0.4	2.9	
Stop breathing during sleep	0.6	0.4	0.1	0.3	0.52		2.4	1.0	0.3	0.2	2.18	
Congested nose at night	2.1	0.8	0.7	0.5	1.38		3.2	0.7	0.4	0.3	2.75	
**Daytime symptoms**												
Tend to breath by mouth during the day	1.2	0.8	0.3	0.2	0.89	0.001	2.5	1.1	0.4	0.3	2.14	0.001
Daytime sleepiness	0.7	0.2	0.4	0.3	0.26	0.03	1.6	1.3	0.8	0.6	0.78	0.001
Difficulty for sustaining attention in tasks	1.7	0.9	1.3	0.7	0.43	0.001	2.3	0.9	1.8	0.5	0.48	0.001
Fail of attention to details	1.8	0.8	1.3	0.7	0.46		2.3	0.8	1.8	0.5	0.51	
Lost of things (toy, pencil...)	1.5	1.3	1.2	1.1	0.31		2.7	1.1	1.9	0.8	0.8	
Easily distracted by extraneous stimuli	1.6	0.9	1.3	0.9	0.29		2.5	0.9	1.8	0.7	0.67	
**Behavior**												
Fidgets with hands or fiting on seat	1.2	1.1	0.9	0.8	0.32	0.001	2.2	1.1	1.5	0.9	0.75	0.001
Leaving seat in classroom	1.0	1.9	0.7	0.7	0.28	0.001	1.9	1.0	1.4	0.8	0.5	0.001
Answersing before question completed	0.9	0.5	0.8	0.7	0.11	0.220	1.5	0.9	1.2	0.9	0.22	0.002
Interrupting or intruding on others	1.4	1.2	1.1	0.9	0.32	0.001	2.0	0.9	1.7	0.8	0.31	0.001
**Respiratory polygraphy**												
AHI, mean ± SD (events/h)	3.9	2.7	0.9	1.0	3.0	0.001	23.4	13.1	3.5	1.4	19.8	<0.001
SpO2, mean ± SD (%)	96.3	2.4	95.5	4.8	0.8	0.244	94.0	7.7	95.2	3.6	1.2	0.060
SpO2 in OSA periode, mean ± SD (%)	86.0	8.8	88.4	7.8	2.6	0.099	74.2	13.9	86.3	9.4	12.1	0.001
Nadir SpO2, mean ± SD (%)	78.8	11.9	83.7	11.8	4.9	0.016	71.9	14.9	81.4	11.3	9.5	0.002
Minimum pulse, mean ± SD (p/min)	56.3	9.6	63.9	23.5	−7.6	0.001	62.6	24.5	62.1	29.0	0.5	0.230
Maximum Pulse, mean ± SD (p/min)	138.5	38.7	121.7	30.8	16.8	0.340	145.8	31.3	125.5	36.3	20.3	0.050
Average pulse, mean ± SD (p/min)	82.8	12.6	70.5	26.2	12.3	0.070	93.8	18.7	89.9	23.5	23.8	0.010
Snoring, mean ± SD (events)	276.6	257.3	154.8	104.2	121.8	0.120	663.6	433.2	221.3	256.4	412.3	0.080

ALR, anti-leukotriene receptor; SG, surgical therapy. ^*^Post vs. pre treatment.

The results of the present study showed that the daytime symptoms due to the consequences of OSA such as breathing by mouth, daytime sleepiness, difficulty sustaining attention in tasks, failure of attention to details, loss of things (toys, pencils...), or easily distracted by extraneous stimuli were improved significantly after treatment with either ALR or ST (Table 3). Other improvements in study subjects' behavior were also recorded after treatment (Table 3).

**Figure 3:**
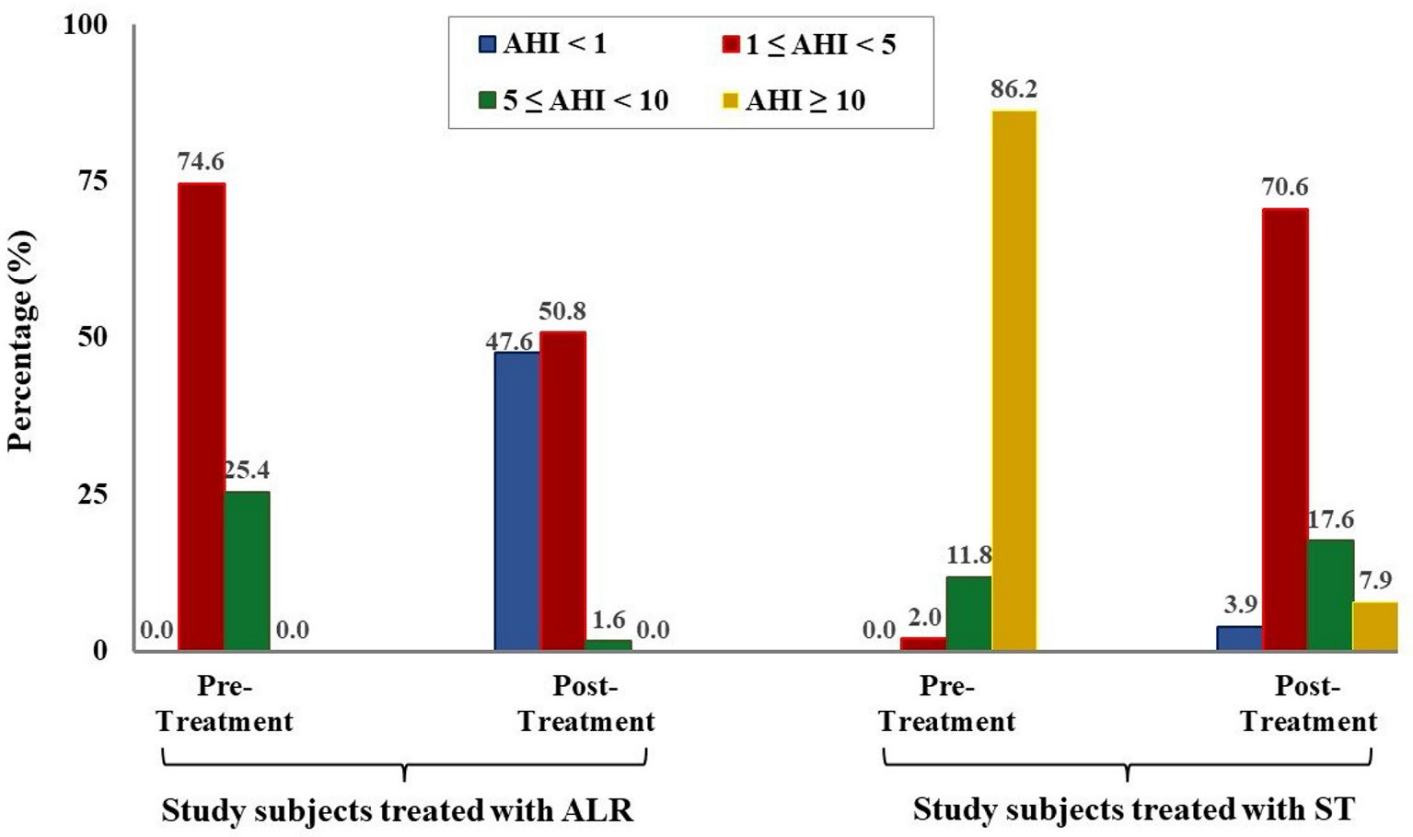
Modification of OSA severity after treatment in study subjects. AHI, apnea-hypopnea index; ALR, anti-leukotriene receptor; SG, surgical therapy.

The results showed that the mean AHI was significantly reduced in comparison with before treatment in study subjects treated with ALR (0.9 ± 1.0 vs. 3.9 ± 2.7 events/h; *p* = 0.001; Table 3) or with ST (3.5 ± 1.4 vs. 23.4 ± 13.1 events/h; *p* < 0.001; Table 3). Nadir SpO2 was significantly improved in study subjects treated with ALR or ST (83.7 ± 11.8 vs. 78.8 ± 11.9% and 81.4 ± 11.3 vs. 71.9 ± 14.9%; *p* = 0.16 and *p* = 0.002; respectively; Table 3). Snoring events were also reduced with LTR treatment or ST vs. before in all study subjects (154.8 ± 104.2 vs. 276.6 ± 257.3 events/h and 221.3 ± 256.4 vs. 663.6 ± 433.2 events/h; *p* = 0.120 and *p* = 0.080; respectively; Table 3). The percentage of study subjects without OSA was significantly increased after being treated with ALR or ST (47.6 vs. 0.0% and 3.9 vs. 0.0%; Figure 3). The percentage of study subjects with moderate OSA treated with ALR was significantly reduced (1.6 vs. 25.4%; Figure 3). The percentage of study subjects with severe OSA was clearly reduced after being treated with ST (7.9 vs. 86.2%; Figure 3).

## Discussion

In the present study, OSA happened mainly in children from 5 to 8 years old and predominantly in boy (Tables 1A,B). Previous studies in both adults and children demonstrated that the prevalence of OSA in men is higher than women; and it might due to the respiratory tract of men are longer than women and more soft structure distribution in the upper respiratory tract (4). Although the percentage of children with low weight is low, there is only few children with overweight or obesity (Table 1). This result is similar with other studies in Asia and different with those done in Western countries where high BMI increases the prevalence of OSA (19, 22, 23). In the present study, there is nearly all children had snoring at night (Table 1). This symptoms might be related to upper airway obstruction caused by tonsillar and/or adenoid hypertrophy. Obviously, the adeno-tonsillar hypertrophy was found mainly in children from 3 to 8 years old with the high rate in the present study (Table 1A). It is similar to the results of other authors and suggest that the main risk for the development of OSAS in young children is hypertrophy of tonsil and adenoid (12, 14).

It is clear that in the present study, the diagnosis of OSA is based on AHI measured by respiratory polygraphy as recommended by guidelines (5, 18, 24). AHI has been defined as the total number of apnea and hypopnea divided by the number of sleeping hours. In children, the cut-off of AHI from 1–5, 5–10 and >10 has been used to refer to mild, moderate, and severe OSA respectively (25). This cut-off is much lower than those in adults (from 5–15, 15–30 and >30). Previous studies demonstrated that children are not mini-adults because many of their physical development processes happening during sleep (18, 24). Therefore, OSA leads to the differences in symptoms, influences and decisions on treatment options between children and adults (12, 14, 24, 25). In the present study, the mean AHI of study subjects is 12.6 ± 11.2 events/ hour and considered as severe OSA. Although, OSA has been well developed in Vietnam in the last 10 years, the early detection of OSA is still difficult, particularly in children. In addition, the costs of respiratory polygraphy or polysomnography are still high and the techniques are time consuming in comparing with other diagnostic tests.

Interestingly, the present study found that there is a significant correlation between the hypertrophy of tonsils and adenoid with the severity of OSA measured by AHI (Figure 2). This result is similar to that reported from other authors (18, 25). Li et al. conducted a study on 1150 children and found that the size of the tonsils was an independent risk factor of OSA (26). The size of the tonsils increased by 25–50% could increase the risk of OSA by two times (OR = 2.0 and *p* = 0.036), the size of the tonsil increased 50–75% could increase the risk of OSA by five times (OR = 5.0 and *p* = 0.022), and if it increased by 75–100%, induced the risk of OSA by eight times (OR = 8.1 and *p* < 0.001).

Beside of that, different studies using universal snoring tools (scales or machines), also revealed the positive correlations between snoring severity (frequency, duration, time, or intensity) and AHI; it was also consistent with the results of present study (data not shown). Moreover, when studying the correlation between nadir SpO_2_ and AHI, the present study found out a significant correlation between two parameters: the more severe of AHI level, the lower level of nadir SpO_2_ (data not shown).

Obviously, the present study shows that the prominent symptoms of OSA were significantly reduced after treatment with ALR and surgical therapy (Table 3). Curiously, in children treated with ALR, the maximum pre- and post-treatment differences (Δmax) of symptoms in the attention deficit and hyperactivity symptom groups (Δmax) were lower than Δmax in the nighttime symptom and daytime symptom groups (Table 3). It might be explained by the apparent improvement in the daytime and nighttime symptom groups related to the fact that ALR reduced the size of tonsil and adenoid hypertrophy and thereby reducing the narrowing of the upper respiratory tract (16, 17). However, in the present study, one children with mild OSA had ST due to increasing symptoms after ALR therapy (Table 2). Among clinical symptoms, morning headache was almost unchanged before and after treatment. This symptom is not common in study children of the present study because there was only 4.8% of them had morning headache (data not shown). But this symptom is quite common in adults with OSA which has been reported by other studies (27, 28). In the present study, children with OSA treated with surgery therapy (adeno-tonsillectomy) also improved significantly their symptoms related to OSA at night and during the day. The largest Δmax was found in symptoms at night and daytime groups while it was lowest for those with attention-reduction and hyperactivity groups (Table 3). Especially, all children with OSA did not have any comorbidities or other current treatments. This result wass similar to previous published reports (12, 25).

**Figure 4:**
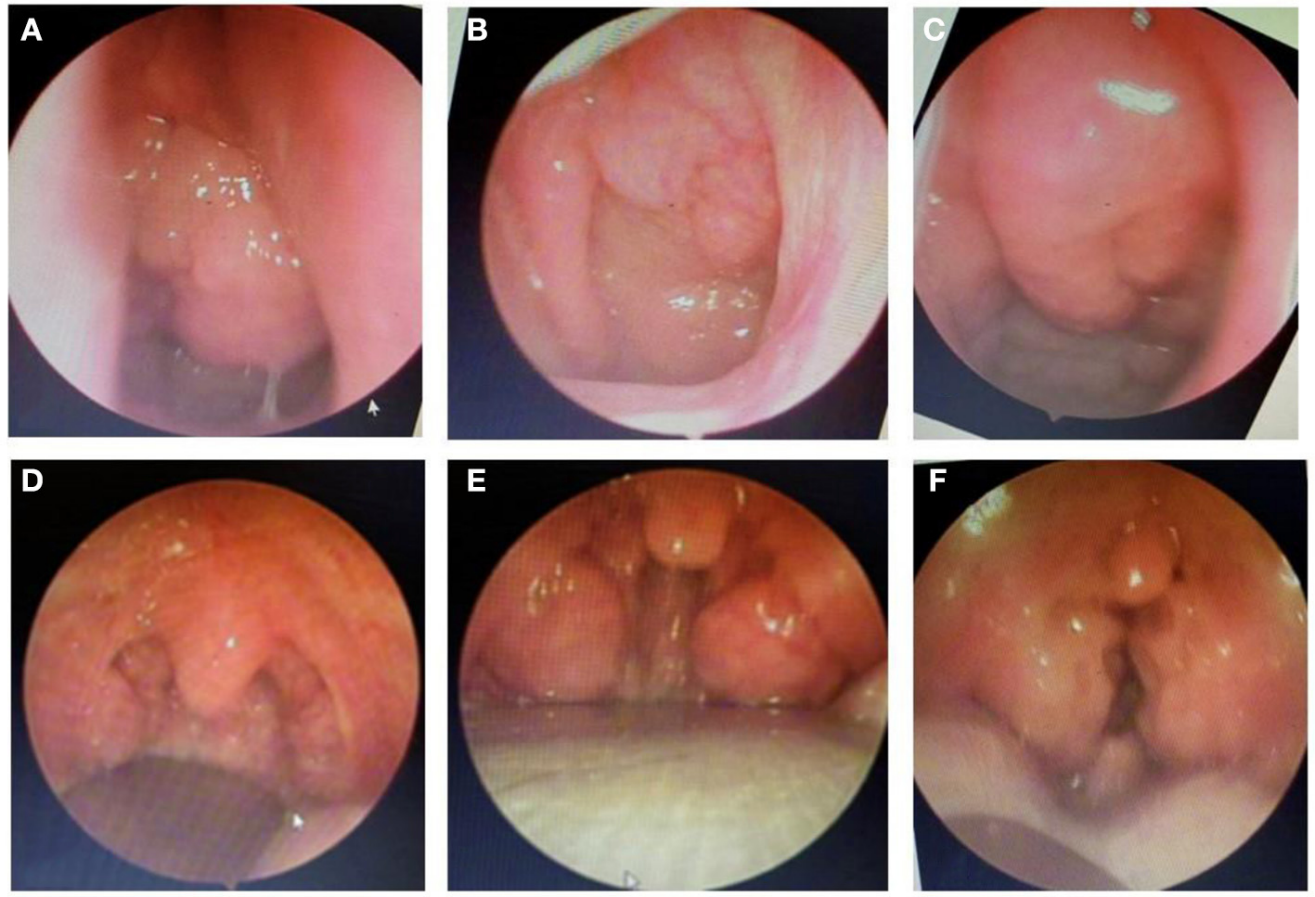
Endoscopic images of adenoid hypertrophy **(A–C)** and tonsillar hypertrophy **(D–F)**.

Finally, the present study demonstrated that after treatment with ALR or ST, the severity of OSA was significantly reduced in study subjects. It is clear that the percentage of children with moderate or severe OSA was reduced by treated with ALR or ST and the mean SpO2 and nadir SpO2 was significantly improved after treatment (Figure 3 and Table 3). These results were similar to previous studies (12, 16, 17, 25). In the present study, surgery therapy seems to be the best effective treatment for children with adenotonsillar hypertrophy associated with or without adenoid hypertrophy (illustrated images from study patients presented in Figure 4) having severe OSA because it improved significantly AHI index and reduced the percentage of severe OSA after interventional procedure (Figure 3). Other works reported the successful rate of treatment for OSA in children with adenotonsillar hypertrophy by surgery method are also very different and depended on centers. The reported successfull rates range from 24 to 100% depending on the study criteria.

Finally, the present study showed that after 12 weeks of ALR treatment, there was no case with side effects requiring discontinuation. Hence, this treatment could be an effective therapy for improving both clinical symptoms and respiratory polygraphy. This medical treatment option might be used as an alternative choice of surgery for adenotonsillar hypertrophy. For study children who underwent adenotonsillar hypertrophy surgery, there was only <10% of reported cases with controlled bleeding during or after the first week of surgery. The main limitation of the present study is related to the short duration of patients' follow-up (three months) with the use of PSQ questionnaires was only 3 months after treatment. Therefore, the long-term follow-up could be necessary for evaluating the significant improvement of recurrent clinical symptoms and hyperactivity and attention deficit in children with OSA.

## Conclusion

OSA is common in children with adenotonsillar hypertrophy. Children with OSA usually have the symptoms at night and its consequences during the day. The severity of adeno-tonsillar hypertrophy is correlated with the severity of OSA measured by apnea-hypopnea index. Fortunately, the treatment of OSA due to adeno-tonsillar hypertrophy with ALR for moderate OSA or surgery for severe OSA can improve children health by reducing nightime and daytime symptoms. However, more studies with long-term follow-up are necessary to evaluate the improvement other daytime consequences of OSA in children with adeno-tonsillar hypertrophy, especially those related to attention decifit and hyperactivity disorders.

## Data availability statement

The raw data supporting the conclusions of this article will be made available by the authors, without undue reservation.

## Ethics statement

The studies involving human participants were reviewed and approved by Ethical Committee of Hanoi Medical University and National Pediatric Hospital (No. 99/HDDD-DHYHN). Written informed consent to participate in this study was provided by the participants' legal guardian/next of kin.

## Author contributions

DT-M, AP-T-Q, PN-D, and SD-Q: conceptualization, methodology, formal analysis, writing–original draft preparation, and writing–review and editing. DT-M, AP-T-Q, and PN-D: software and validation. All authors contributed to the article and approved the submitted version.

## Conflict of interest

The authors declare that the research was conducted in the absence of any commercial or financial relationships that could be construed as a potential conflict of interest. The reviewer TNNP declared a shared secondary affiliation with the author SDQ to the handling editor at the time of review.

## Publisher's note

All claims expressed in this article are solely those of the authors and do not necessarily represent those of their affiliated organizations, or those of the publisher, the editors and the reviewers. Any product that may be evaluated in this article, or claim that may be made by its manufacturer, is not guaranteed or endorsed by the publisher.
